# International Medical Congress

**Published:** 1881-10

**Authors:** 


INTERNATIONAL MEDICAL CONGRESS.—So far as we have been able to learn, the recent international medical meeting in London was a very satisfactory one, both in regard to the number of delegates in attendance and the quantity and quality of work done. The arrangements appear to have been well made for transacting the proper business of meeting, while the hospitalities extended were abundant and cordial, and the number of those present from the various nations on both sides of the Atlantic was sufficient to give it a more truly international character than any of the congresses that had preceded it. We have been on the lookout for some items concerning the important work done there by the numerous delegates representing our own city, but our looking has thus far been in vain. We must be content, therefore, until they come home and report for themselves.

